# Estradiol Enhances Alveolar Bone Resorption by Promoting Osteoclast Differentiation in Experimental Periodontitis

**DOI:** 10.3390/dj14070420

**Published:** 2026-07-09

**Authors:** Keisuke Yasuda, Shinji Matsuda, Takumi Memida, Tetsuya Yoshimoto, Fuminori Nakashima, Yurika Ninomiya, Tomoya Ueda, Shogo Shimada, Shizu Hirata-Tsuchiya, Mikihito Kajiya, Kazuhisa Ouhara, Noriyoshi Mizuno

**Affiliations:** 1 Department of Periodontal Medicine, Graduate School of Biomedical and Health Sciences, Hiroshima University, Hiroshima 734-8553, Japanmizuno@hiroshima-u.ac.jp (N.M.); 2 Division of Periodontology, Department of Regenerative Science in Conservative Dentistry, Kyushu Dental University, Kitakyushu 803-8580, Japan; 3 Center of Oral Clinical Examination, Hiroshima University Hospital, Hiroshima 734-8551, Japan; 4 Department of Oral Science and Translation Research, College of Dental Medicine, Nova Southeastern University, Fort Lauderdale, FL 33314, USA; 5 Health Promotion Section, Health Services Bureau, Hiroshima 730-8586, Japan

**Keywords:** ovariectomy, anastrozole, estrogen, osteoclasts, periodontitis

## Abstract

**Background/Objectives**: Estrogen is a key female hormone; however, its role in periodontitis remains poorly understood. This study investigated the effects of 17β-estradiol (E2) on experimental periodontitis using an ovariectomy (OVX) model with E2 administration. **Methods**: Female mice aged 8–10 weeks underwent OVX, followed by induction of ligature-induced periodontitis, and subsequent quantification of alveolar bone resorption. Additional groups received an aromatase inhibitor or E2 supplementation after OVX, with subsequent induction of periodontitis and evaluation of bone resorption. Histological analysis assessed multinucleated giant cells and tartrate-resistant acid phosphatase-positive osteoclasts on the bone surface. Gingival tissue was analyzed for gene expression related to osteoclastogenesis. The effect of E2 on osteoclast differentiation from bone marrow cells was also examined. **Results**: OVX significantly reduced serum E2 levels and decreased alveolar bone resorption. Aromatase inhibitor administration similarly reduced bone loss. Histological evaluation revealed a reduced number of resorbing osteoclasts in OVX mice, whereas E2 supplementation increased osteoclast numbers. No significant changes in inflammatory cytokine or receptor activator of nuclear factor-kappa B ligand (RANKL) expression were observed. E2 promoted osteoclast differentiation in vitro, and treatment with E2 prior to RANKL stimulation further increased the number of osteoclasts. This effect was suppressed by an estrogen receptor antagonist. Moreover, E2 enhanced the expression of osteoclast differentiation–associated genes in the presence of RANKL, an effect abolished by tamoxifen. **Conclusions**: E2 increased alveolar bone resorption in experimental periodontitis, likely by promoting osteoclast differentiation, independent of inflammatory cytokine or RANKL gene expression.

## 1. Introduction

Recent advances in research on female health have highlighted the importance of sex-specific factors in disease susceptibility and progression. Although many health challenges are shared between males and females, the physiological roles of female sex hormones, particularly estrogen, warrant focused investigation. The effects of estrogen on periodontal tissues have been reviewed extensively [1], with its impact believed to vary depending on age, hormonal status, and systemic background.

Clinical observations suggest that estrogen may modulate periodontal inflammation. Gingival inflammation reportedly exacerbates during adolescence [2] and menstruation [3], and the development of gingivitis and periodontitis during pregnancy [4,5] further supports a hormonal influence. Conversely, estrogen deficiency following menopause is a well-established risk factor for osteoporosis [6], and the use of estrogen inhibitors in postmenopausal patients with breast cancer is associated with an elevated risk of periodontitis [7]. However, the effects of estrogen replacement therapy on periodontal disease remain controversial, with studies reporting both beneficial [8] and negligible outcomes [9].

These conflicting findings underscore the need for a more precise understanding of estrogen’s role in inflammation-induced alveolar bone destruction. Therefore, the present study aimed to elucidate the effects of 17β-estradiol (E2) on alveolar bone resorption using a murine model of experimental periodontitis, combining ovariectomy (OVX)-induced E2 deficiency with exogenous E2 administration.

## 2. Materials and Methods

### 2.1. Animal Experiments

All experiments were conducted using the minimum number of animals necessary, with sample size calculated using the G*Power software (version 3.1.9.7) (α = 0.05, power = 0.8 or 0.9). Female wild-type (WT) and C57BL/6J mice aged 8–12 weeks were purchased from Japan Clea (Tokyo, Japan) and housed under a 12-h light/dark cycle (lights on at 8:00 a.m., off at 8:00 p.m.) at 22 ± 2 °C with ad libitum access to food and water. Mice were randomly assigned to the experimental groups.

OVX was performed as described previously [10]. A small flank incision (3–5 mm) was made under aseptic conditions to expose the ovaries, which were ligated at the fallopian tubes and excised. To minimize the influence of endogenous ovarian hormones, all animals underwent ovariectomy prior to estradiol administration. E2 administration followed established protocols [11]. Moreover, E2 pellets (SE-121, Innovative Research of America, Sarasota, FL, USA) were implanted via a 0.5 cm incision in the neck skin, with a subcutaneous pocket created laterally using blunt dissection. A sham-operated group served as the control for E2 pellet implantation, in which a neck skin incision was made in OVX mice without E2 pellet insertion.

As previously reported [12], experimental periodontitis was induced by silk ligation of the second maxillary molar 30 days after OVX and maintained for 1 week. To enable direct comparison between groups under comparable inflammatory conditions, ligatures were applied to all experimental animals. The period for OVX was determined according to previous reports, which demonstrated that bone metabolism changes become evident at 4 weeks after OVX in both the jawbone and femur [13,14]. The ligature period was set to 7 days based on prior studies [15], as severe alveolar bone resorption occurs by this time, with no further increase thereafter. Therefore, in this study, the ligature was applied for 7 days. A shorter ligature duration limits the detection of differences in bone resorption. E2 was administered concurrently with OVX, and a sham-operated group (neck incision only) was included. To assess the impact of E2 synthesis inhibition, WT mice received anastrozole (200 μg/kg/day, 11987; Cayman Chemical, Arbor, MI, USA) for 1 week, followed by ligature-induced periodontitis for 1 week, as described previous report [16]. Serum E2 levels ere measured using an enzyme-linked immunosorbent assay kit (KGE014, R&D Systems, Minneapolis, MN, USA) by Hokudo (Hokkaido, Japan) to verify the reduction in endogenous hormone levels after ovariectomy.

### 2.2. Evaluation of Alveolar Bone Resorption

#### Micro-Computed Tomography (CT) Analysis

Imaging was performed using CosmoScan GXIII (Rigaku, Tokyo, Japan) at 100 kV, 120 μA, 25.8 μm/pixel resolution, and 25.8 μm slice thickness. Alveolar bone resorption between the maxillary first and second molars was quantified as previously reported [17]. To minimize inter-sample variation, regions with clearly visible bifurcation of the second molar were selected. For micro-CT analysis, two measurement points were obtained from each specimen, and all values were included in the analysis. Therefore, the reported values represent measurement points rather than the number of animals. Bone resorption was measured as the distance from the cementoenamel junction (CEJ) to the alveolar bone crest (ABC) along the distal root of the first molar and the mesial root of the second molar. ImageJ software (version 1.54p) was employed for measurements.

Sagittal sections spanning the first to third molars included ~50 layers, with layer 1 corresponding to the palatal crown of the first molar and layer 50 to the buccal mesial root. Representative images were obtained from around layer 30.

Furthermore, three-dimensional analysis of alveolar bone was performed using CTAn software (Bruker) (version 1.12.0.0 +). Bone volume/total volume (BV/TV) was calculated within a defined region of interest (ROI) in the palatal alveolar bone adjacent to the second molar, corresponding to the area of ligature-induced bone loss. The ROI was defined based on anatomical landmarks, including the cementoenamel junction (CEJ) and alveolar bone crest (ABC), to ensure consistent evaluation across samples. In addition, bone loss volume (%) was defined as 100 − BV/TV (%) within the same ROI.

### 2.3. Histological Evaluation

Maxillary bones were decalcified in 10% ethylenediaminetetraacetic acid for 1 week, dehydrated in ethanol and xylene, and embedded in paraffin. Serial 7 μm sections were cut in the buccal-palatal plane and stained with hematoxylin-eosin (HE) and tartrate-resistant acid phosphatase (TRAP). Histomorphometric analysis was performed using an optical microscope with ×10 eyepiece and ×4 or ×10 objective lenses.

Alveolar bone resorption was assessed within a 500 μm × 500 μm area in sagittal sections encompassing the distal roots of the first and second molars. The CEJ–ABC distance was measured along the mesial root of the second molar. Among the serial sections, after the furcation was identified, three to six sections at 100-μm intervals were averaged to obtain a single value for each animal, and three animals per group were utilized for statistical analysis. Additionally, multinucleated giant cells and TRAP-positive cells on the bone surface within a 500 μm × 500 μm area beneath the furcation of the second molar were counted as previously described [18].

### 2.4. Gene Expression Analysis

Messenger ribonucleic acid (mRNA) expression in the gingival tissue was analyzed as previously described [19]. Mice were euthanized, and palatal gingival tissue was excised and stored in RNAlater (r0901, Sigma-Aldrich, St. Louis, MO, USA) at 4 °C for 24 h. Total RNA was extracted using the RNeasy Fibrous Tissue Mini Kit (QIAGEN, Valencia, CA, USA). Additionally, RNA from cultured cells was isolated using RNAiso Plus (TaKaRa Bio, Shiga, Japan).

Complementary DNA was synthesized using 500 ng of RNA with Sensiscript Reverse Transcriptase (QIAGEN, Valencia, CA, USA), LightCycler, and SYBR Green. Polymerase chain reaction was performed with initial denaturation at 95 °C for 10 min, followed by 40 cycles of 95 °C for 15 s and 60 °C for 1 min. Gene expression was quantified using the ΔΔCt method and normalized to glyceraldehyde 3-phosphate dehydrogenase. Primer sequences are listed in Supplementary Table S1.

### 2.5. Evaluation of the Number of Osteoclasts

Bone marrow mesenchymal cells were isolated from the tibiae and femurs of 13–15-week-old female mice. After erythrocyte depletion, the cells were cultured in alpha minimum essential medium supplemented with 25 ng/mL macrophage colony-stimulating factor (M-CSF; 576404, BioLegend, San Diego, CA, USA) for 10 days to induce macrophage differentiation. Osteoclastogenesis was induced by co-stimulation with M-CSF and 100 ng/mL receptor activator of nuclear factor-κB ligand (RANKL; 769404, BioLegend, San Diego, CA, USA) for 7–10 days. β-Estradiol (10 nM, E2758, Sigma-Aldrich, St. Louis, MO, USA) or tamoxifen (10 nM, 13258, Cayman Chemical, Arbor, MI, USA) stimulation followed the schedule and concentration displayed in Supplementary Figure S1. To determine the optimal estradiol concentration for osteoclast differentiation, bone marrow-derived macrophages were treated with estradiol at concentrations of 0, 0.1, 1, 10, 100, and 1000 nM. Based on this dose–response analysis, 10 nM estradiol was selected for subsequent experiments (Supplementary Figure S2). Cells were fixed in 10% formalin and stained using the TRAP/ALP staining kit (294-67001, FUJIFILM WAKO, Osaka, Japan). TRAP-positive multinucleated cells were counted as osteoclasts.

### 2.6. Statistical Analysis

Statistical analyses were performed using JMP Pro version 18 (JMP Statistical Discovery LLC, NC, USA). For comparisons among three or more groups, Tukey’s multiple comparison test or Dunnett’s test was used for normally distributed data. In contrast, the Steel–Dwass test was employed for non-normally distributed data. Comparisons between two groups were conducted using Student’s *t*-test or the Mann–Whitney U test. A significance level of α = 0.05 was applied throughout the experiment. All experiments were conducted with biological replicates. Different analyses required independent specimens; therefore, sample sizes varied across experiments depending on the type of analysis. Parametric analyses were applied to the in vitro data, whereas non-parametric analyses were used for the in vivo data. Sample size estimation was performed using JMP software based on a two-sample comparison. The significance level (α) was set at 0.05 and the statistical power was set at 0.8, with alveolar bone resorption (exposed root length) defined as the primary outcome, as previously described [20].

## 3. Results

### 3.1. Effects of OVX and Anastrozole Administration on Alveolar Bone Resorption in Experimental Periodontitis

**Figure 1:**
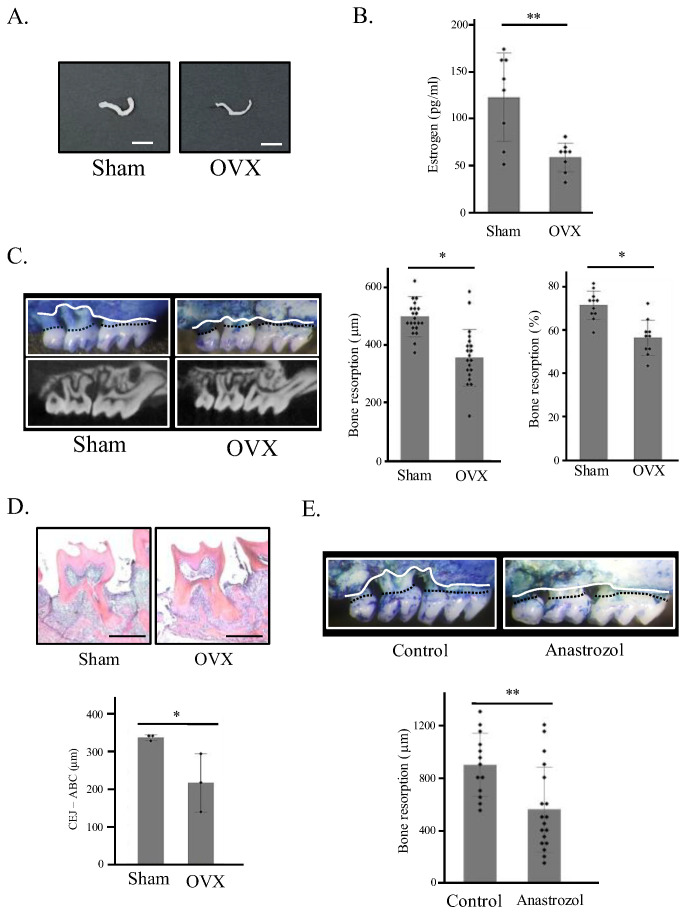
Effects of ovariectomy (OVX) and aromatase inhibition on ligature-induced periodontitis. (**A**) Representative image displaying uterine shrinkage following OVX. Scale bar = 10 mm. (**B**) Serum 17β-estradiol (E2) levels in sham-operated and OVX mice (sham [n = 8], OVX [n = 8]). ** *p* < 0.01. (**C**) Evaluation of bone resorption in experimental periodontitis after OVX. Upper panels: visual assessment using Giemsa staining. The white line indicates alveolar bone crest (ABC). The black dotted line indicates the cementoenamel junction (CEJ). Lower panels: micro-computed tomography images. Central Panel: Quantification of the distance from the cementoenamel junction to the alveolar bone crest (sham [n = 21], OVX [n = 20]). Right panel: Quantification of bone loss volume (%) (sham [n = 11], OVX [n = 10]), * *p* < 0.05 (**D**) Histological evaluation of alveolar bone resorption (sham [n = 3], OVX [n = 3]). Scale bar = 500 μm. * *p* < 0.05. (**E**) Effect of aromatase inhibitor (anastrozole) on ligature-induced periodontitis. Representative Giemsa-stained images (control [n = 13], anastrozole [n = 18]). ** *p* < 0.01.

Uterine atrophy and reduced serum E2 levels were confirmed after OVX, consistent with the outcomes of previous reports [21] (Figure 1A,B). Alveolar bone resorption was quantified following OVX to assess the impact of E2 deficiency on periodontitis-induced bone destruction. The CEJ–ABC distance was significantly decreased in OVX mice compared with in WT controls, indicating decreased bone loss (Figure 1C). Histological analysis corroborated these findings, demonstrating a marked decrease in bone resorption in OVX mice (Figure 1D). Similarly, administration of the aromatase inhibitor anastrozole in WT mice significantly decreased alveolar bone resorption (Figure 1E).

### 3.2. Effects of E2 Administration on Alveolar Bone Resorption

**Figure 2:**
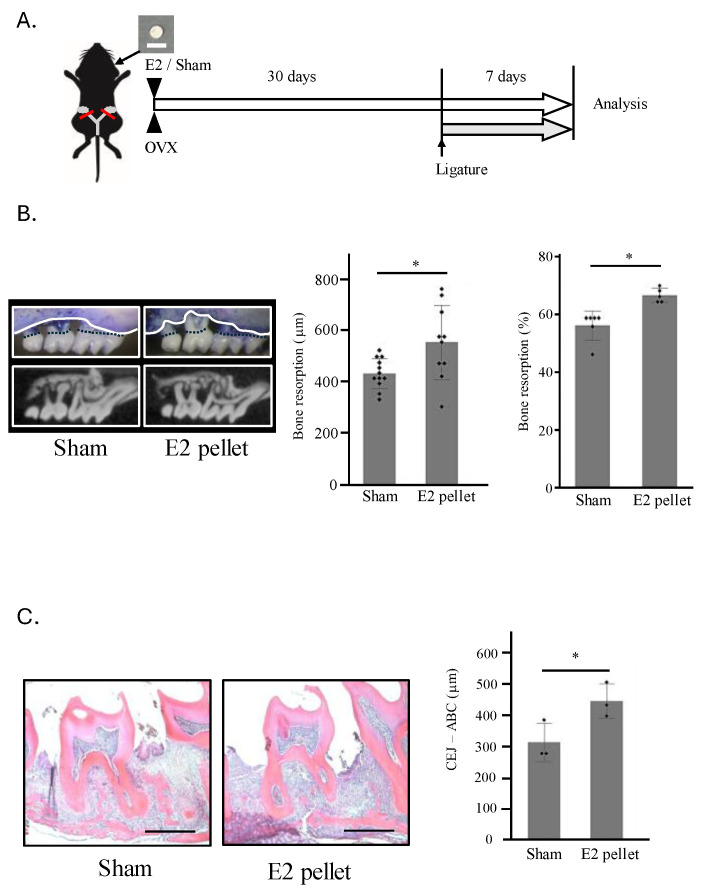
Role of 17β-estradiol (E2) in ligature-induced periodontitis in ovariectomy (OVX) mice. (**A**) Experimental schedule. (**B**) Evaluation of bone resorption in OVX mice with or without E2 pellet administration. The sham operation group (Sham) underwent a neck skin incision without E2 pellet implantation in OVX mice. The group with E2 pellets implanted in the neck skin is referred to as the “E2 pellets” group. Upper panels: Giemsa-stained images. Lower panels: micro-computed tomography images. The white line indicates alveolar bone crest (ABC). The black dotted line indicates the cementoenamel junction (CEJ). Central panel: Quantification of cementoenamel junction–alveolar bone crest distance (sham [n = 12], E2 pellet [n = 10]). Right panel: Quantification of bone loss volume (%) (sham [n = 6], E2 pellet [n = 5]) * *p* < 0.05. (**C**) Histological evaluation of alveolar bone resorption (sham [n = 3], E2 pellet [n = 3]). Scale bar = 500 μm. * p < 0.05.

To determine whether the OVX-induced reduction in bone resorption was E2-dependent, exogenous E2 was administered to OVX mice, and alveolar bone resorption was evaluated (Figure 2A). E2 supplementation physiologically increased the serum estradiol concentration to an average of 72.4 pg/mL. Moreover, E2 supplementation reversed the OVX-induced decrease in bone resorption and significantly increased bone resorption (Figure 2B). Histological evaluation further revealed that E2 pellet administration increased bone loss from the CEJ to the alveolar crest, compared with that in untreated OVX mice (Figure 2C).

### 3.3. Histopathological Evaluation of E2-Related Bone Resorption

**Figure 3:**
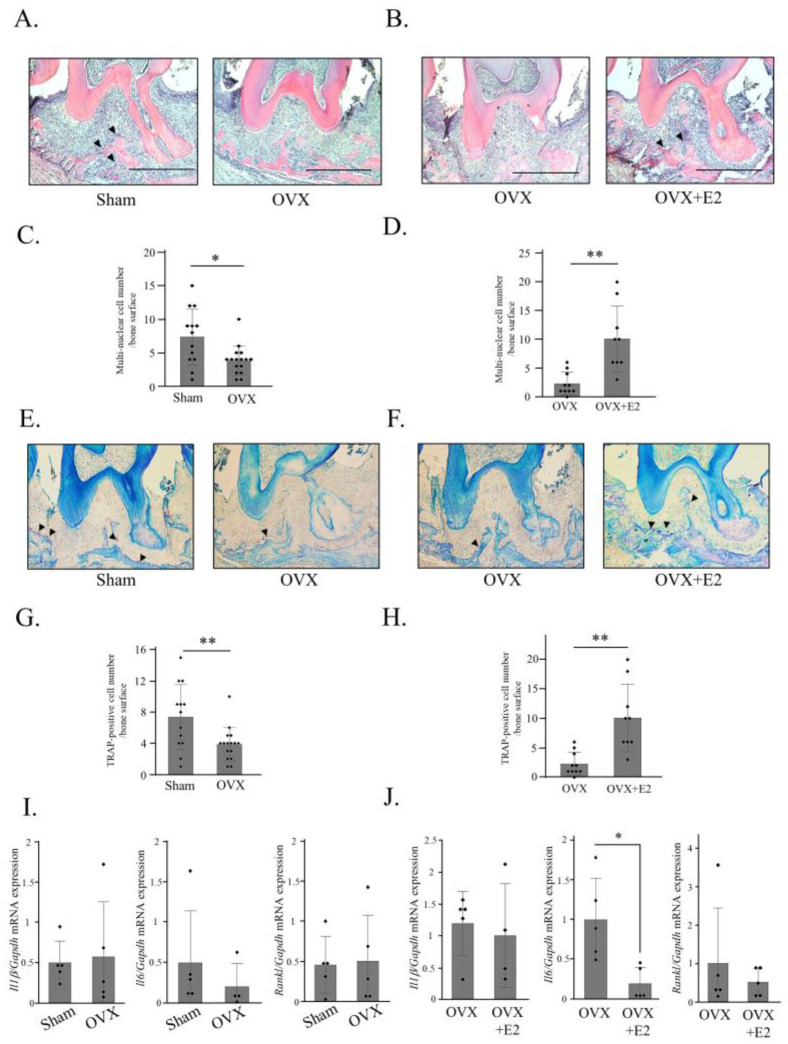
17β-estradiol (E2)-mediated osteoclastogenesis in ligature-induced periodontitis. (**A**,**B**) Hematoxylin and eosin-stained images displaying multinucleated giant cells on the bone surface in ovariectomy (OVX) mice (**A**) and OVX mice with E2 pellet administration (**B**). The black arrow points to multinucleated giant cells. Scale bar = 500 μm. (**C**,**D**) Quantification of multinucleated giant cells (sham [n = 13], OVX [n = 16]) (OVX [n = 10], OVX + E2 [n = 9]). * *p* < 0.05, ** *p* < 0.01. (**E**,**F**) Tartrate-resistant acid phosphatase (TRAP)-stained images demonstrating osteoclasts on the bone surface in sham and OVX mice (**E**), and OVX mice with or without E2 pellet administration (**F**). The black arrow points to TRAP-positive cells. (**G**,**H**) Quantification of TRAP-positive cells (sham [n = 13], OVX [n = 16]) (OVX [n = 10], OVX + E2 [n = 9]). ** *p* < 0.01. (**I**,**J**) Messenger ribonucleic acid expression of osteoclast-related genes in the gingival tissue from OVX and E2-treated mice. Expression levels of interleukin (IL)1β, IL6, and receptor activator of nuclear factor-κB ligand (n = 5/group). * *p* < 0.05.

Histological analysis was performed to investigate the cellular mechanisms underlying E2-mediated bone resorption. OVX mice exhibited a marked reduction in the number of multinucleated giant cells compared with those in non-OVX controls (Figure 3A,C). In contrast, E2 administration increased the number of multinucleated giant cells (Figure 3B,D). TRAP staining revealed a significant decrease in the number of TRAP-positive osteoclasts in OVX mice (Figure 3E,G), while E2 supplementation restored the number of osteoclasts (Figure 3F,H). Despite these changes, gene expression analysis of inflammatory cytokines and RANKL in gingival tissue revealed no significant differences between OVX and non-OVX groups, and E2 administration did not alter their expression (Figure 3I,J).

### 3.4. Evaluation of the Effect of E2 on Osteoclast Differentiation

**Figure 4:**
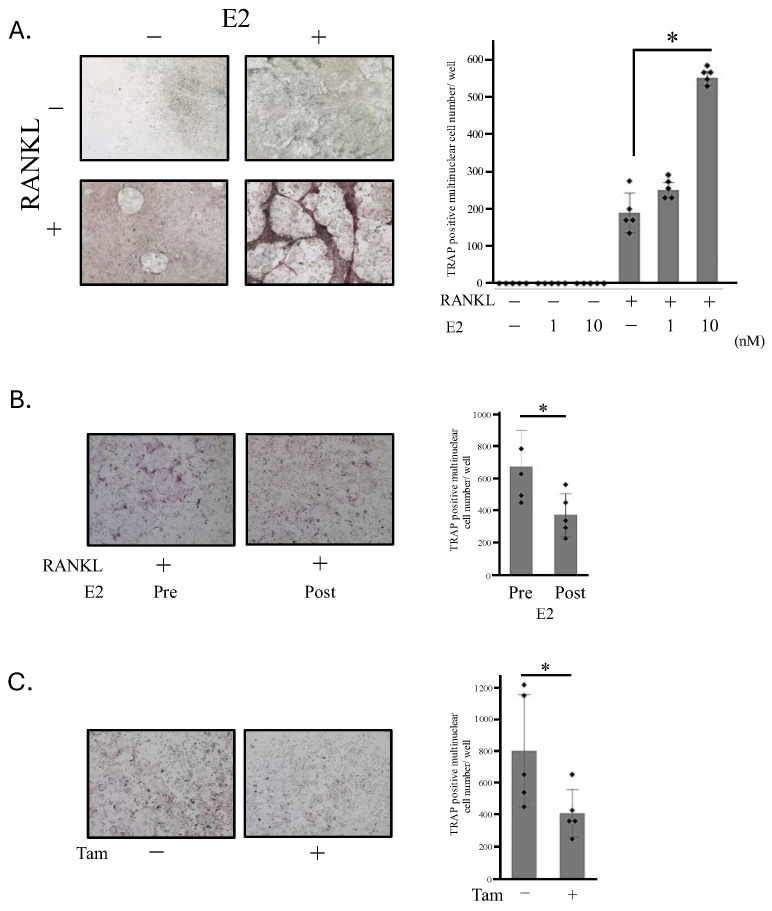
The role of 17β-estradiol (E2) in osteoclast differentiation. (**A**) Tartrate-resistant acid phosphatase-stained images of multinucleated osteoclasts with or without E2 treatment (RANKL − E2 − [n = 5], RANKL − E2 1 nM [n = 5], RANKL − E2 10 nM [n = 5], RANKL+ E2 − [n = 5], RANKL+ E2 1 nM [n = 5], RANKL+ E2 10 nM [n = 5]). * *p* < 0.05. (**B**) Effect of pre- or post- treatment with E2 on osteoclast differentiation (Pre E2 [n = 4], Post E2 [n = 5]). * *p* < 0.05. (**C**) Effect of tamoxifen, an estrogen receptor antagonist, administered 24 h before E2 stimulation (Tam − [n = 5], Tam + [n = 5]). * *p* < 0.05.

Given the lack of change in inflammatory cytokine and RANKL expression, the direct effect of E2 on osteoclast differentiation was examined. E2 stimulation was applied according to the schedule illustrated in Supplementary Figure S1. Co-stimulation with E2 and RANKL significantly increased the number of osteoclasts (Figure 4A). Interestingly, E2 administration before RANKL stimulation enhanced osteoclastogenesis, whereas post- RANKL stimulation E2 treatment reduced the number of osteoclasts (Figure 4B); these effects were suppressed by tamoxifen, a selective estrogen receptor antagonist, as confirmed in macrophages [22,23] (Figure 4C). To elucidate the mechanisms by which estrogen enhances osteoclast formation, we evaluated the mRNA expression of genes related to osteoclast differentiation from the early stage to maturation [24].

**Figure 5:**
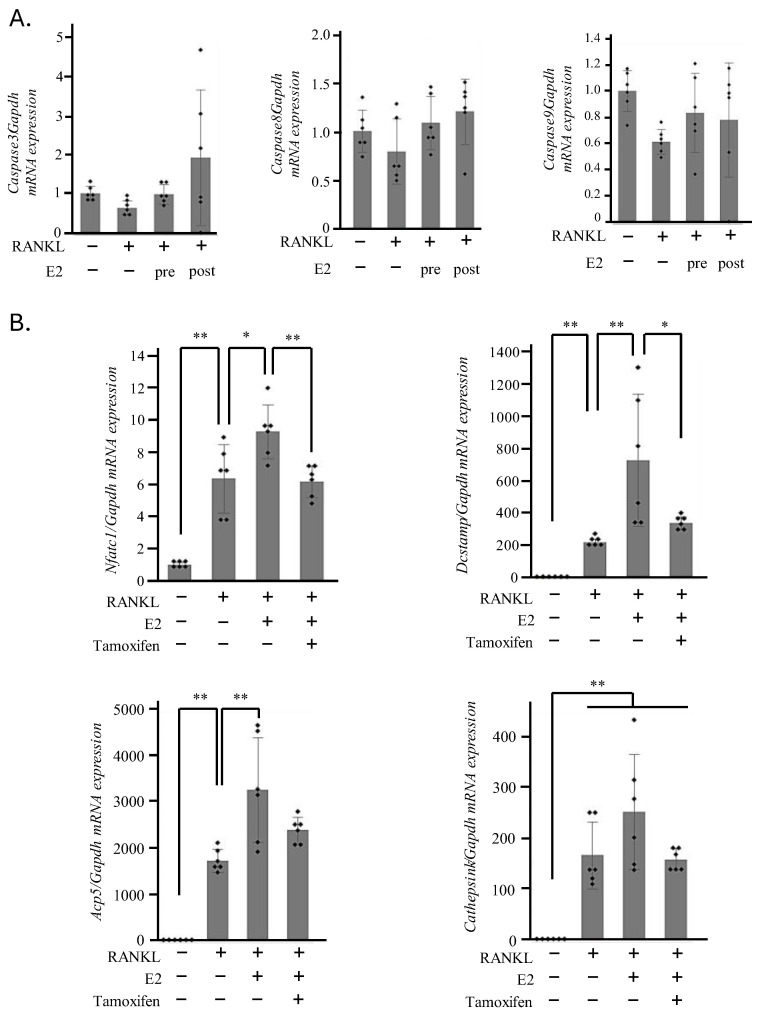
The role of 17β-estradiol (E2) in messenger ribonucleic acid (mRNA) expressions related to apoptosis and osteoclast differentiation. (**A**) Caspase 3, 8, 9 mRNA expressions in osteoclasts with E2 pre- or post-treatment were analyzed. Schedule was the same as Supplementary Figure S1 (C) (receptor activator of nuclear factor-κB ligand [RANKL] − E2 − [n = 6], RANKL + E2 − [n = 6], RANKL + Pre E2 [n = 6], RANKL + Post E2 [n = 6]). (**B**) Osteoclasts differentiation associated genes, *Nfatc1*, *DC-stamp*, *Cathepsin k* and *Acp5* in osteoclasts with or without E2 and tamoxifen were analyzed. Schedule was same as Supplementary Figure S1 (D) (RANKL − E2 − Tam − [n = 6], RANKL+ E2− Tam − [n = 6], RANKL + E2 + Tam − [n = 6], RANKL + E2 + Tam + [n = 6)]. * *p* < 0.05, ** *p* < 0.01.

In addition, we examined whether estradiol induces osteoclast apoptosis, as previously reported among its effects on osteoclasts [25]. Post- or pre-stimulation with E2 after RANKL application did not alter the expression of apoptosis-related genes, including caspase-3, -8, and -9, compared with the control (Figure 5A). However, E2 stimulation increased the expression levels of NFATc1 and DC-STAMP, with the increase inhibited by tamoxifen (Figure 5B).

## 4. Discussion

This study provides the first evidence that E2 enhances osteoclast activity and increases alveolar bone resorption in experimental periodontitis. Notably, in this study, E2 increased the number of osteoclasts by stimulating macrophages prior to osteoclast differentiation in an estrogen receptor-dependent manner.

Sex differences in periodontal disease are multifactorial, with both males and females exhibiting distinct risk profiles [26]. Biological effects of sex hormones differ between sexes and vary across life stages. Although one study identified no significant association between serum estrogen levels and periodontitis in adult females [27], hormone replacement therapy for postmenopausal estrogen deficiency has been reported to suppress periodontitis-induced bone resorption [8]. However, clinical comparisons between postmenopausal and pregnant or menstruating females are confounded by differences in age, diet, and other background factors, making the isolation of estrogen’s specific effects difficult.

To address this, we employed a controlled experimental model to evaluate the role of E2 in periodontitis at the molecular level. OVX was used to induce E2 deficiency. Previous studies reported conflicting results: some discovered no impact of OVX on periodontitis-induced bone loss [28], whereas others observed enhanced bone resorption following long-term OVX [29]. These discrepancies may stem from differences in the timing of evaluation after OVX, as prolonged E2 deficiency can cause osteoporosis and altered bone properties. In our study, evaluation was performed 4 weeks after OVX—an interval sufficient to confirm E2 reduction while minimizing confounding effects from bone remodeling. The present study was designed to evaluate the role of estradiol under uniform inflammatory conditions induced by ligature placement in all experimental groups, rather than to examine all combinations of OVX and ligation status.

We confirmed uterine atrophy and reduced serum E2 levels following OVX. OVX suppressed alveolar bone resorption induced by silk ligation, as evidenced by both micro-CT and histological analyses. To further validate the role of E2, we administered an aromatase inhibitor, anastrozole, which reduced the serum E2 levels in mice and humans [30,31] and suppressed bone resorption consistent with the OVX model.

To investigate the effects of E2 supplementation, we administered E2 pellets in OVX mice. E2 has been reported to exacerbate asthma [32] and alleviate psoriasis [33], indicating that its role in inflammation remains tissue- and context-dependent. In our model, E2 administration exacerbated experimental periodontitis, suggesting that its pro-inflammatory or pro-resorptive effects may be specific to periodontal tissues or the ligature-induced inflammatory mechanism.

Histological evaluation revealed that OVX reduced the number of multinucleated giant cells and TRAP-positive osteoclasts, whereas E2 administration increased both cell types. These findings suggest that E2 promotes osteoclast differentiation, contributing to increased bone resorption. Osteoclasts have been reported to be induced in experimental periodontitis, spreading not only near the silk filament, but also throughout the entire alveolar bone, consistent with our findings [34]. To explore the underlying mechanism, we examined the expression of key genes involved in osteoclastogenesis, including interleukin (IL)-1β [35], *IL-6* [36], *RANKL* [37], and osteoprotegerin (*OPG*), a decoy receptor of RANK. Surprisingly, no significant changes in gene expression were observed after OVX or E2 treatment, indicating that E2 may not modulate osteoclast-related gene expression in the gingival tissue. Furthermore, OPG was not detected in periodontal tissue with or without OVX or estradiol administration.

We therefore evaluated the direct effect of E2 on osteoclast differentiation in vitro. Bone marrow-derived macrophages were stimulated with M-CSF and RANKL, with E2 administered at various time points. E2 enhanced osteoclast differentiation when applied prior to RANKL stimulation but suppressed differentiation when administered after RANKL exposure. This phase-dependent effect was abolished by tamoxifen, implicating estrogen receptor-mediated signaling during the macrophage-to-osteoclast precursor transition. Previous reports indicate that E2 increases Fas ligand expression in osteoclast precursor cells and mature osteoclasts, directly inducing osteoclast apoptosis [38]. However, other reports indicate that E2 does not affect mature osteoclasts [39], leaving uncertainties regarding the phase of cell differentiation and the mechanism by which E2 reduces osteoclasts. Furthermore, the process of inducing differentiation from bone marrow cells to osteoclasts involves heterogeneous cells. Additionally, only a subset of these cells is believed to differentiate into mature osteoclasts [40]. This study’s results suggest that applying low E2 concentrations during the undifferentiated stage of osteoclasts promotes osteoclast differentiation. Osteoclast differentiation was enhanced in bone marrow cells treated with E2 before RANKL administration, whereas administering E2 after initiating RANKL treatment suppressed osteoclast differentiation. Furthermore, E2-stimulated osteoclast differentiation was suppressed by tamoxifen, an estrogen receptor antagonist, suggesting that E2 stimulation during the differentiation of macrophages to osteoclast precursor cells is involved in estrogen receptor-mediated osteoclast differentiation. Previous studies evaluating E2-induced macrophage transcription and function reported that E2 modulated gene expression related to the cell cycle and stimulated cell proliferation [41]. Furthermore, another report indicated that the number of pre-differentiated cells in bone marrow or macrophages influenced osteoclast differentiation [42], supporting the interpretation of the present study’s findings. In this study, bone marrow cells were stimulated with M-CSF for a longer duration before RANKL exposure than in previous reports, which used 30 ng/mL M-CSF for 4 days before RANKL stimulation [43]. Another study reported the use of CD11c-positive cells isolated from bone marrow before RANKL stimulation [44]. These methodological differences from the current study may account for the discrepancies observed in osteoclast formation. This study has certain limitations. Osteoclast differentiation was assessed using mouse-derived cells; future studies using human peripheral blood monocytes would improve clinical relevance. Furthermore, we did not evaluate periodontitis in a long-term OVX model with established osteoporosis, nor did we investigate extended periods of ligation. Severe bone resorption may occur 7 days after ligation; however, changes beyond this time point were not assessed in the present study. This study focused on the early phase of ligature-induced alveolar bone resorption, which has been reported to reach a plateau within approximately 7 days in mice [15]. Investigating how osteoporotic bone changes influence periodontal inflammation could provide insights into disease mechanisms in postmenopausal females. In addition, analyses were not performed blinded, and incorporating blinding may have been necessary for optimization.

In the current study, together with previous reports, estrogen was identified to regulate osteoclast fate in a manner dependent on cell heterogeneity. Prolonged M-CSF stimulation of bone marrow cells may promote macrophage and pre-osteoclast proliferation and heterogeneity via cell-cycle activation [45,46], accompanied by increased mRNA expression of differentiation-related genes (*NFATc1* and *DC-STAMP*) [24] and osteoclast-maturation genes (*cathepsin K* and *Acp5*) [24], as observed in our findings. After RANKL exposure, E2 suppresses osteoclastogenesis by activating apoptotic pathways, including ERα-dependent FasL signaling and caspase cascades (caspase-8 and caspase-9), together with mitochondrial Bax/Bak activity [39], which collectively dismantle mature osteoclasts and limit bone resorption. The dynamics of ERα expression explain these timing-specific effects [47], clarifying why E2 can both protect bone and exacerbate inflammation-driven resorption. To the best of our knowledge, this study is the first to demonstrate that females possess an active immune response against infection as a self-defense mechanism, with estrogen thought to contribute to this process [48]. Periodontitis triggered by an activated immune response may serve as a self-defense mechanism by promoting the loss of infected teeth [49], with E2 playing a potential role in this process.

## 5. Conclusions

This study demonstrates that short-term E2 reduction suppresses experimental periodontitis, whereas E2 administration promotes alveolar bone resorption. The underlying mechanism appears to involve estrogen receptor-mediated activation of macrophages, enhancing osteoclast induction. These findings contribute to a deeper understanding of hormonal regulation in periodontal disease and may inform future therapeutic strategies.

## Data Availability

The original contributions presented in this study are included in the article/Supplementary Materials. Further inquiries can be directed to the corresponding authors.

## Supplementary Materials

The following supporting information can be downloaded at: https://www.mdpi.com/article/10.3390/dj14070420/s1. Figure S1: Experimental schedule. Figure S2: The dose-dependent analysis of estradiol treatment on bone marrow-derived macrophages. Table S1: Primer sequences used in this study. Table S2: The characteristics of patients with periodontal disease (PD) and drug-induced gingival enlargement (DIGE).

## Abbreviations

The following abbreviations are used in this manuscript:

E2	17 β-estradiol
OVX	ovariectomy
RANKL	receptor of activator nuclear factor-kappa B ligand
CEJ	cemento-enamel junction
ABC	alveolar bone crest
HE	hematoxylin-eosin
TRAP	tartrate-resistant acid phosphatase

## References

[1] Sathish A.K., Varghese J., Fernandes A.J. (2022). The impact of sex hormones on the periodontium during a Woman’s lifetime: A concise-review update. Curr. Oral Health Rep..

[2] Nakagawa S., Fujii H., Machida Y., Okuda K. (1994). A longitudinal study from prepuberty to puberty of gingivitis. Correlation between the occurrence of *Prevotella intermedia* and sex hormones. J. Clin. Periodontol..

[3] Khosravisamani M., Maliji G., Seyfi S., Azadmehr A., Abd Nikfarjam B., Madadi S., Jafari S. (2014). Effect of the menstrual cycle on inflammatory cytokines in the periodontium. J. Periodontal Res..

[4] Thomas C., Timofeeva I., Bouchoucha E., Canceill T., Champion C., Groussolles M., Arnaud C., Vayssière C., Nabet C., Laurencin-Dalicieux S. (2023). Oral and periodontal assessment at the first trimester of pregnancy: The PERISCOPE longitudinal study. Acta Obstet. Gynecol. Scand..

[5] Tilakaratne A., Soory M., Ranasinghe A.W., Corea S.M.X., Ekanayake S.L., De Silva M. (2000). Periodontal disease status during pregnancy and 3 months post-partum, in a rural population of Sri-Lankan women. J. Clin. Periodontol..

[6] Väänänen H.K., Härkönen P.L. (1996). Estrogen and bone metabolism. Maturitas.

[7] Taichman L.S., Inglehart M.R., Giannobile W.V., Braun T., Kolenic G., Van Poznak C. (2015). Periodontal health in women with early-stage postmenopausal breast cancer newly on aromatase inhibitors: A pilot study. J. Periodontol..

[8] Man Y., Zhang C., Cheng C., Yan L., Zong M., Niu F. (2024). Hormone replacement therapy and periodontitis progression in postmenopausal women: A prospective cohort study. J. Periodontal Res..

[9] Ageel R., Abaalkhail B., Natto Z.S. (2025). Effect of hormone replacement therapy on periodontal health in post-menopausal women in Jeddah, Saudi Arabia. BMC Womens Health.

[10] Luengo-Mateos M., González-Vila A., Torres Caldas A.M., Alasaoufi A.M., González-Domínguez M., López M., González-García I., Barca-Mayo O. (2024). Protocol for ovariectomy and estradiol replacement in mice. STAR Protoc..

[11] Ingberg E., Theodorsson A., Theodorsson E., Strom J.O. (2012). Methods for long-term 17β-estradiol administration to mice. Gen. Comp. Endocrinol..

[12] Hatano S., Matsuda S., Okanobu A., Furutama D., Memida T., Kajiya M., Ouhara K., Fujita T., Mizuno N., Kurihara H. (2021). The role of nuclear receptor 4A1 (NR4A1) in drug-induced gingival overgrowth. FASEB J..

[13] Li Z., Kuhn G., Schirmer M., Müller R., Ruffoni D. (2017). Impaired bone formation in ovariectomized mice reduces implant integration as indicated by longitudinal in vivo micro-computed tomography. PLoS ONE.

[14] Zhou S., Wang G., Qiao L., Ge Q., Chen D., Xu Z., Shi D., Dai J., Qin J., Teng H. (2018). Age-dependent variations of cancellous bone in response to ovariectomy in C57BL/6J mice. Exp. Ther. Med..

[15] Kittaka M., Yoshimoto T., Levitan M.E., Urata R., Choi R.B., Teno Y., Xie Y., Kitase Y., Prideaux M., Dallas S.L. (2023). Osteocyte RANKL drives bone resorption in mouse ligature-induced periodontitis. J. Bone Miner. Res..

[16] Xanthopoulos J.M., Romano A.E., Majumdar S.K. (2005). Response of mouse breast cancer cells to anastrozole, tamoxifen, and the combination. J. Biomed. Biotechnol..

[17] Nakashima F., Matsuda S., Ninomiya Y., Ueda T., Yasuda K., Hatano S., Shimada S., Furutama D., Memida T., Kajiya M. (2024). Role of sclerostin deletion in bisphosphonate-induced osteonecrosis of the jaw. Bone.

[18] Simão M., Camacho A., Ostertag A., Cohen-Solal M., Pinto I.J., Porto G., Hang Korng E., Cancela M.L. (2018). Iron-enriched diet contributes to early onset of osteoporotic phenotype in a mouse model of hereditary hemochromatosis. PLoS ONE.

[19] Ueda T., Matsuda S., Ninomiya Y., Nakashima F., Yasuda K., Furutama D., Memida T., Yoshimoto T., Kajiya M., Ohta K. (2024). Nuclear receptor 4A1 (NR4A1) upregulated by n-butylidenephthalide via the mitogen-activated protein kinase (MAPK) pathway ameliorates drug-induced gingival enlargement. BioFactors.

[20] Chen Z., Zhong Y., Chen L., Liu W., Lin C., Chen Y., Wang X. (2025). HGF Aggravated Periodontitis Associated Gut Barrier and Microbial Dysfunction: Implications for Oral–Gut Axis Regulation. Biology.

[21] Yousefzadeh N., Kashfi K., Jeddi S., Ghasemi A. (2020). Ovariectomized rat model of osteoporosis: A practical guide. EXCLI J..

[22] Murphy A.J., Guyre P.M., Wira C.R., Pioli P.A. (2009). Estradiol regulates expression of estrogen receptor ERα46 in human macrophages. PLoS ONE.

[23] Yu W., Zheng H., Lin W., Tajima A., Zhang Y., Zhang X., Zhang H., Wu J., Han D., Rahman N.A. (2014). Estrogen promotes Leydig cell engulfment by macrophages in male infertility. J. Clin. Investig..

[24] Takegahara N., Kim H., Choi Y. (2024). Unraveling the intricacies of osteoclast differentiation and maturation: Insight into novel therapeutic strategies for bone-destructive diseases. Exp. Mol. Med..

[25] Saintier D., Khanine V., Uzan B., Ea H.K., de Vernejoul M.C., Cohen-Solal M.E. (2006). Estradiol inhibits adhesion and promotes apoptosis in murine osteoclasts in vitro. J. Steroid Biochem. Mol. Biol..

[26] Ioannidou E. (2017). The sex and gender intersection in chronic periodontitis. Front. Public Health.

[27] Su X., Jin K., Zhou X., Zhang Z., Zhang C., Li Y., Yang M., Huang X., Xu S., Wei Q. (2023). The association between sex hormones and periodontitis among American adults: A cross-sectional study. Front. Endocrinol..

[28] Dai J., Ma Y., Shi M., Cao Z., Zhang Y., Miron R.J. (2016). Initial changes in alveolar bone volume for sham-operated and ovariectomized rats in ligature-induced experimental periodontitis. Clin. Oral Investig..

[29] Amadei S.U., Souza D.M., Brandão A.A.H., da Rocha R.F. (2011). Influence of different durations of estrogen deficiency on alveolar bone loss in rats. Braz. Oral Res..

[30] Wang W., Belosay A., Yang X., Hartman J.A., Song H., Iwaniec U.T., Turner R.T., Churchwell M.I., Doerge D.R., Helferich W.G. (2016). Effects of letrozole on breast cancer micro-metastatic tumor growth in bone and lung in mice inoculated with murine 4T1 cells. Clin. Exp. Metastasis.

[31] Gupta P.B., Kuperwasser C. (2006). Contributions of estrogen to ER-negative breast tumor growth. J. Steroid Biochem. Mol. Biol..

[32] Warren K.J., Deering-Rice C., Huecksteadt T., Trivedi S., Venosa A., Reilly C., Sanders K., Clayton F., Wyatt T.A., Poole J.A. (2023). Steady-state estradiol triggers a unique innate immune response to allergen, resulting in increased airway resistance. Biol. Sex. Differ..

[33] Adachi A., Honda T., Egawa G., Kanameishi S., Takimoto R., Miyake T., Hossain M.R., Komine M., Ohtsuki M., Gunzer M. (2022). Estradiol suppresses psoriatic inflammation in mice by regulating neutrophil and macrophage functions. J. Allergy Clin. Immunol..

[34] Wu Y.-H., Taya Y., Kuraji R., Ito H., Soeno Y., Numabe Y. (2020). Dynamic microstructural changes in alveolar bone in ligature-induced experimental periodontitis. Odontology.

[35] Bloemen V., Schoenmaker T., De Vries T.J., Everts V. (2011). IL-1β favors osteoclastogenesis via supporting human periodontal ligament fibroblasts. J. Cell. Biochem..

[36] Feng W., Liu H., Luo T., Liu D., Du J., Sun J., Wang W., Han X., Yang K., Guo J. (2017). Combination of IL-6 and sIL-6R differentially regulates varying levels of RANKL-induced osteoclastogenesis through NF-κB, ERK, and JNK signaling pathways. Sci. Rep..

[37] Mo S., Jang J.S., Lee S.H., Kim H.H. (2024). Single-cell transcriptome analysis reveals periodontal ligament fibroblast heterogeneity with distinct IL-1β and RANKL expression in periodontitis. Mol. Cells.

[38] Novack D.V. (2007). Estrogen and bone: Osteoclasts take center stage. Cell Metab..

[39] Kim H.N., Ponte F., Nookaew I., Ucer Ozgurel S., Marques-Carvalho A., Iyer S., Warren A., Aykin-Burns N., Krager K., Sardao V.A. (2020). Estrogens decrease osteoclast number by attenuating mitochondrial oxidative phosphorylation and ATP production in early osteoclast precursors. Sci. Rep..

[40] Tsukasaki M., Takayanagi H. (2022). Osteoclast biology in the single-cell era. Inflamm. Regen..

[41] Pepe G., Braga D., Renzi T.A., Villa A., Bolego C., D’Avila F., Barlassina C., Maggi A., Locati M., Vegeto E. (2017). Self-renewal and phenotypic conversion are the main physiological responses of macrophages to the endogenous estrogen surge. Sci. Rep..

[42] Ikeda K., Takeshita S. (2016). The role of osteoclast differentiation and function in skeletal homeostasis. J. Biochem..

[43] Marques-Carvalho A., Sardão V.A., Kim H.N., Almeida M. (2023). ECSIT is essential for RANKL-induced stimulation of mitochondria in osteoclasts and a target for the anti-osteoclastogenic effects of estrogens. Front. Endocrinol..

[44] Srivastava S., Toraldo G., Weitzmann M.N., Cenci S., Ross F.P., Pacifici R. (2001). Estrogen decreases osteoclast formation by down-regulating receptor activator of NF-κB ligand (RANKL)-induced JNK activation. J. Biol. Chem..

[45] Nasser H., Adhikary P., Abdel-Daim A., Noyori O., Panaampon J., Kariya R., Okada S., Ma W., Baba M., Takizawa H. (2020). Establishment of bone marrow-derived M-CSF receptor-dependent self-renewing macrophages. Cell Death Discov..

[46] Hamilton T.A., Zhao C., Pavicic P.G., Datta S. (2014). Myeloid colony-stimulating factors as regulators of macrophage polarization. Front. Immunol..

[47] Oreffo R.O.C., Kusec V., Virdi A.S., Flanagan A.M., Grano M., Zambonin-Zallone A., Triffitt J.T. (1999). Expression of estrogen receptor-alpha in cells of the osteoclastic lineage. Histochem. Cell Biol..

[48] Cutolo M., Sulli A., Straub R.H. (2012). Estrogen metabolism and autoimmunity. Autoimmun. Rev..

[49] Tsukasaki M., Komatsu N., Nagashima K., Nitta T., Pluemsakunthai W., Shukunami C., Iwakura Y., Nakashima T., Okamoto K., Takayanagi H. (2018). Host defense against oral microbiota by bone-damaging T cells. Nat. Commun..

