# Supplementary material for: Estradiol Enhances Alveolar Bone Resorption by Promoting Osteoclast Differentiation in Experimental Periodontitis
Source: Dent J (Basel). 2026 Jul 9;14(7):420. doi: 10.3390/dj14070420 (PMC13409665; doi:10.3390/dj14070420)
Supplement: Supplementary file 1 [file dentistry-14-00420-s001.zip › Supplementary Figure S1 final proof.pdf]

Fig.S1

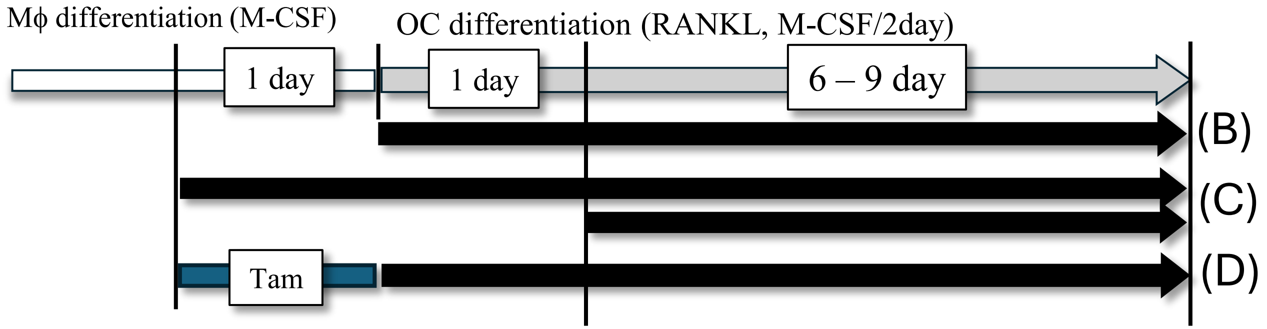

Fig.S1 Experimental schedule.
After macrophage differentiation induced by macrophage colony-stimulating factor, receptor activator of nuclear factor-κB ligand (RANKL) was applied. E2 stimulation was administered according to the indicated schedule.
