# Supplementary material for: Estradiol Enhances Alveolar Bone Resorption by Promoting Osteoclast Differentiation in Experimental Periodontitis
Source: Dent J (Basel). 2026 Jul 9;14(7):420. doi: 10.3390/dj14070420 (PMC13409665; doi:10.3390/dj14070420)
Supplement: Supplementary file 1 [file dentistry-14-00420-s001.zip › Supplementary Figure S2 final proof.pdf]

Fig.S2

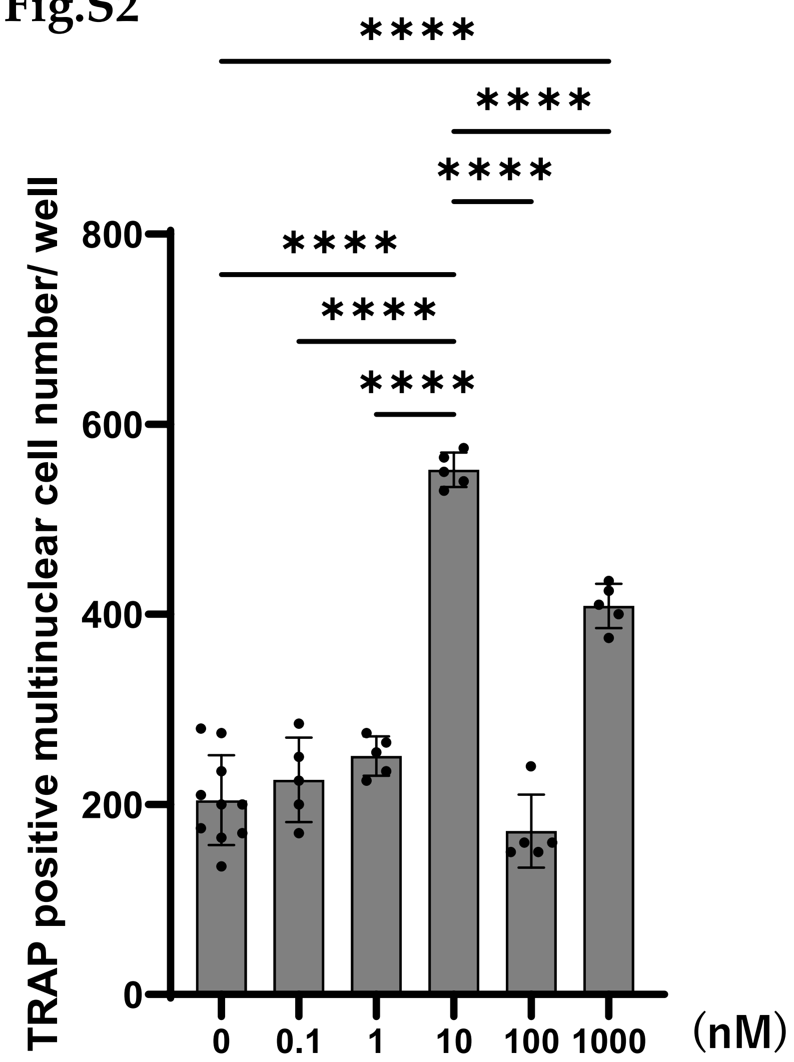

Fig.S2 The dose-dependent analysis for estradiol treatment to bone marrow-derived macrophages.
Bone marrow-derived macrophages were treated with estradiol at concentrations of 0, 0.1, 1, 10, 100, and 1000 nM. Osteoclast formation was most prominently increased at 10 nM estradiol.
The x-axis shows the concentration of estradiol.
The statistical analysis is used one-way ANOVA. ****: $p<0.0001$
