# Supplementary material for: Estradiol Enhances Alveolar Bone Resorption by Promoting Osteoclast Differentiation in Experimental Periodontitis
Source: Dent J (Basel). 2026 Jul 9;14(7):420. doi: 10.3390/dj14070420 (PMC13409665; doi:10.3390/dj14070420)
Supplement: Supplementary file 1 [file dentistry-14-00420-s001.zip › Supplementary Tables S1 and S2.pdf]

Table S1. Primer sequences used in this study.

Mice		
Gapdh	Forward	CCTGGAGAAACCTGCCAAGTATG
	Reverse	TGTTGCTGTAGCCGTATTCATTGT
Il1b	Forward	CTGGTACATCAGCACCTCACA
	Reverse	GAGCTCCTTAACATGCCCTG
Il6	Forward	GAAATGAGAAAAGAGTTGTGCAATGG
	Reverse	ATATCCAGTTTGGTAGCATCCATCAT
Rankl	Forward	CATCCCATCGGGTTCCCATAA
	Reverse	CCTTAGTTTTCCGTTGCTTAACGAC
Caspase3	Forward	GGAGTCTGACTGGAAAGCCGAA
	Reverse	CTTCTGGCAAGCCATCTCCTCA
Caspase8	Forward	ATGGCTACGGTGAAGAACTGCG
	Reverse	TAGTTCACGCCAGTCAGGATGC
Caspase9	Forward	GCTGTGTCAAGTTTGCCTACCC
	Reverse	CCAGAATGCCATCCAAGGTCTC
NFATc1	Forward	GGTGCCTTTTGCGAGCAGTATC
	Reverse	CGTATGGACCAGAATGTGACGG
DC-Stamp	Forward	TTTGCCGCTGTGGACTATCTGC
	Reverse	GCAGAATCATGGACGACTCCTTG
ACP5	Forward	GCGACCATTGTTAGCCACATACG
	Reverse	CGTTGATGTCGCACAGAGGGAT
Cathepsin K	Forward	AGCAGAACGGAGGCATTGACTC
	Reverse	CCCTCTGCATTTAGCTGCCTTTG

Table S2. The characteristics of patients with periodontal disease (PD) and drug-induced gingival enlargement (DIGE).

PD	age	sex	Stage	Grade	DIGE	age	sex	drug	disease	*p*-value (age)	*p*-value (sex)
1	77	F	II	B	1	34	M	ciclosporin	Aplastic anemia	0.61	0.65
2	58	F	III	B	2	71	F	amlodipine	Hypertension		
3	81	M	I	A	3	69	M	phenytoin	Epilepsy		
4	40	F	III	C	4	66	F	ciclosporin	Aplastic anemia		
5	74	M	I	A	5	57	F	nifedipine	Hypertension		
6	57	F	III	C	6	81	F	amlodipine	Hypertension		
7	51	M	III	B	7	51	M	phenytoin	Epilepsy		
8	80	F	II	A	8	47	M	amlodipine	Hypertension		
9	76	F	II	A	9	59	F	amlodipine	Hypertension		
10	55	F	IV	C	10	80	M	amlodipine	Hypertension		

F; female, M; male
